# Supplementary material for: Nasal Mucosa Exploited by SARS-CoV-2 for Replicating and Shedding during Reinfection
Source: Viruses. 2022 Jul 23;14(8):1608. doi: 10.3390/v14081608 (PMC9394478; doi:10.3390/v14081608)
Supplement: Supplementary file 1 [file viruses-14-01608-s001.zip › viruses-1792481-supplementary.pdf]

Nasal mucosa exploited by SARS-COV-2 for replicating and shedding during reinfection

Heng Li[a #], Xin Zhao[a #], Jing Li[a #], Huiwen Zheng[a #], Yurong Zhao[a], Jinling Yang[a], Jingxian Zhou[a], Fengmei Yang[a], YanliChen[a], Yuanyuan Zuo[a], Qingrun Lai[a], Haiting Long[a], Yanyan Li[a], Weihua Jin[a], Haijing Shi[a], Longding Liu[a] *

a Institute of Medical Biology, Chinese Academy of Medical Sciences & Peking Union Medical College, Kunming 650118, China

#These authors contributed equally to this work.

*Corresponding author: Longding Liu, E-mail: longdingl@gmail.com.

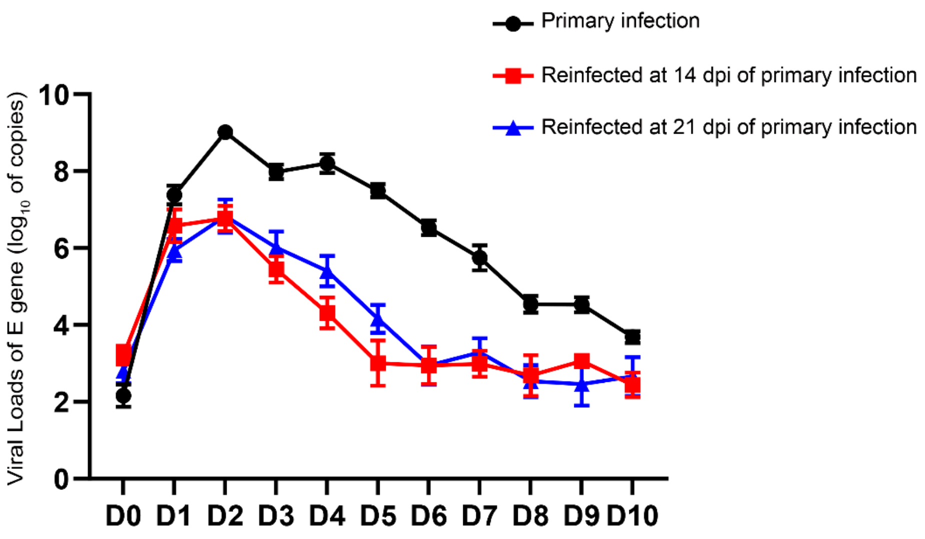

Figure S1. Viral shedding in 1 ml nasal washes from 0 dpi to 10 dpi in the primary infection and reinfection.

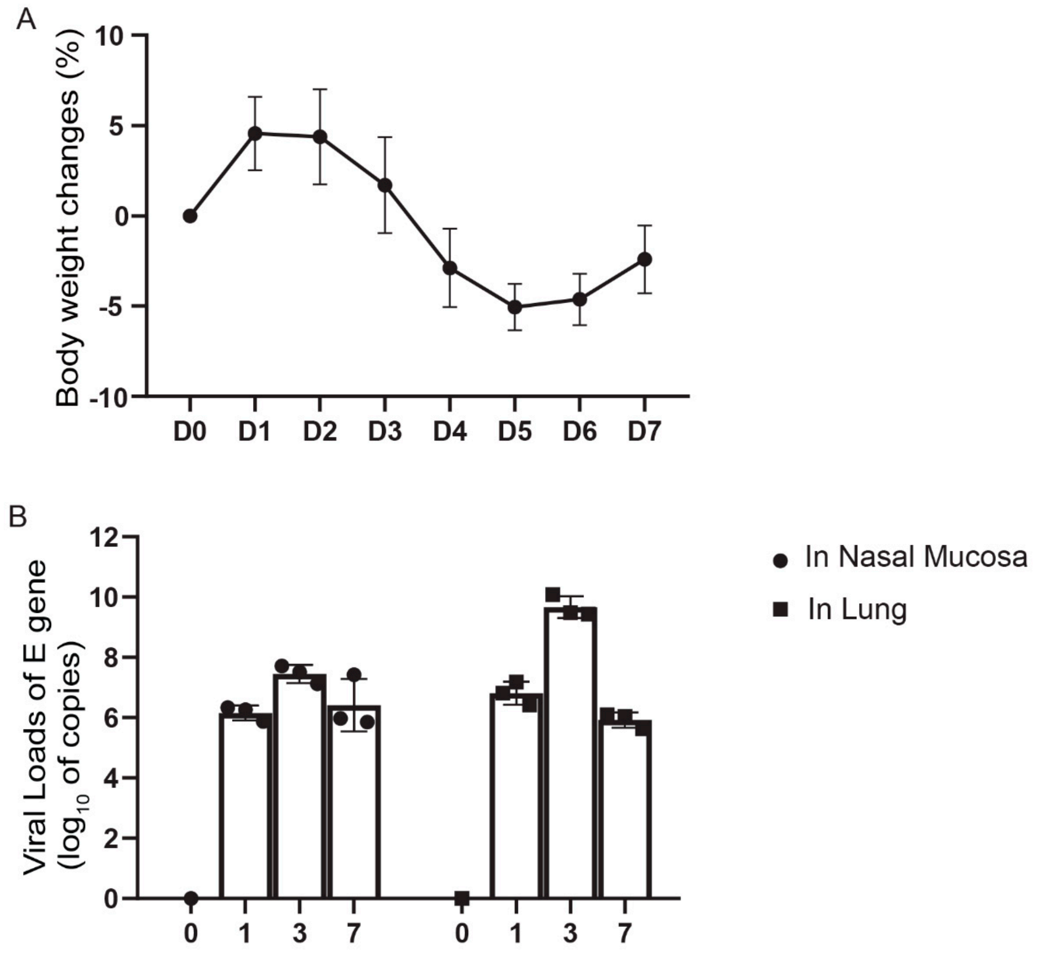

Figure S2. The infectivity in healthy Syrian hamsters of the shedding from upper respiratory tract in the primary infection and reinfection.

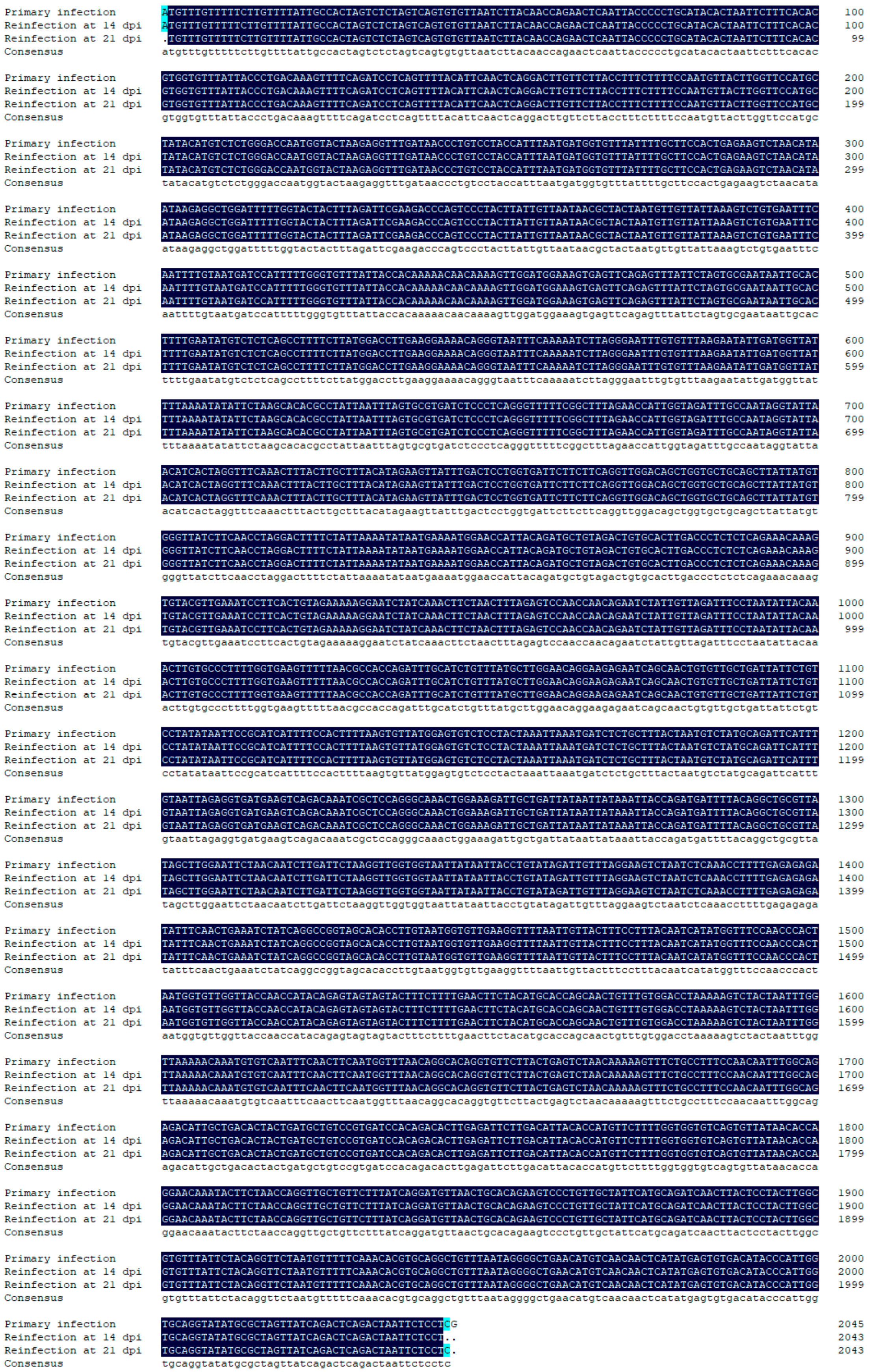

Figure S3: No difference in the S1 gene sequence from the nasal washes between the

primary infection and reinfection.

Table S1. *p*-value among viral loads in various tissues in the primary and secondary infection by SPSS PASW statistical software version 18.0

			Viral load in nose	Viral load in nasal mucosa	Viral load in lung	Viral load in trachea	Viral load in bulbus olfactorius	Viral load in cerebrum	Viral load in jaw	Viral load in NALF	Viral load in BALF	sgRNA in nose	sgRNA in nasal mucosa
D0	A	B	0.000	0.000	0.000							0.386	
		C	0.000	0.000	0.000							0.001	
	B	C	0.360	0.000	0.014							0.001	
D3	A	B	0.000	0.000	0.000	0.000	0.000	0.000	0.081	0.000	0.000	0.019	0.155
		C	0.000	0.000	0.000	0.000	0.000	0.000	0.005	0.000	0.000	0.010	0.007
	B	C	0.863	0.891	0.001	0.003	0.362	0.380	0.061	0.066	0.473	0.592	0.054
D5	A	B	0.000	0.000	0.000	0.000	0.000	0.000	0.009	0.000	0.000	0.000	0.000
		C	0.000	0.000	0.000	0.004	0.000	0.000	0.001	0.000	0.000	0.000	0.000
	B	C	0.007	0.006	0.080	0.038	0.236	0.000	0.037	0.000	1.000	0.219	0.000
D7	A	B	0.000	0.000	0.000	0.000	0.000	0.000	0.005	0.003	0.001	0.000	0.000
		C	0.000	0.000	0.000	0.014	0.000	0.001	0.003	0.002	0.000	0.000	0.000
	B	C	0.006	0.698	0.050	0.002	0.210	0.279	0.747	0.703	0.783	0.297	1.000
D10	A	B	0.000	0.009	0.000	0.001	0.037	0.038	0.043		0.037	0.558	0.000
		C	0.000	0.001	0.000	0.061	0.007	0.035	0.023		0.004	0.063	0.000
	B	C	0.071	0.089	0.015	0.010	0.237	0.952	0.646		0.113	0.027	1.000

A Primary infection; B Reinfected at 14 dpi of primary infection; C Reinfected at 21 dpi of primary infection

NALF were nasal pharyngeal larvage fluid, BALF were bronchoalveolar larvage fluid.

Table S2. Histology score standards of the lung damage

**Categories**	**Score**
Pulmonary parenchymal	0 none<br>1 The lung structure was clear, and the alveolar septum was slightly thickened, very few inflammatory cells infiltrate<br>2 Local edema, local alveolar septum thickening, alveolar cells had slightly exfoliated necrosis, a small amount of inflammatory cell infiltration<br>3 Some alveolar septum was thickened, accompanied by mild parenchymal lesions, a small amount of alveolar cell degeneration necrosis, moderate amount of inflammatory cell infiltration<br>4 Large area of the alveolar septum was thickened, some substantial lesions, large area of alveolar structure has disappeared, alveolar

	cells have degeneration and necrosis, a large number of inflammatory cell infiltration<br>5 Lung structure disappeared with extensive consolidation and diffuse infiltration of inflammatory cells
Bronchus and Bronchiole	0 none<br>1 Local tracheal epithelium was slightly diseased with slight cell abscission<br>2 Part of trachea epithelial cells were shed, and mild amount of exudate was observed in the lumen<br>3 The cells were denaturated and necrotic, part of the mucosa is detached, and a large number of inflammatory exudates and tissue fragments could be seen in the lumen<br>4 The cells were denaturated and necrotic, and a large number of mucosa detached, part of the lumen was blocked<br>5 The cells were denaturated and necrotic, and a large number of the mucosa was detached, A large area or the total lumen was blocked
Pulmonary vasculitis	0 none<br>1 Mild edema and hyperemia, minimal inflammatory cell infiltrated around the lumen<br>2 Vascular edema, local endothelial cells were necrotic, a small number of inflammatory cell infiltration<br>3 Endothelial cells were denaturated and necrotic with vascular wall hyperplasia, inflammatory cell infiltration<br>4 Vascular wall cells were denaturated and necrotic, with lumen stenosis and inflammatory cell infiltration<br>5 Vascular structures were ruptured or many cells were denaturated and necrotic, diffuse infiltration of inflammatory cells

Table S3. Sequences of primers for real time RT-qPCR detection of mRNA gene expression of host cytokines

Gene name	Forward primer (5’ to 3’)	Reverse Primer (5’ to 3’)
β-actin	ATGGCCAGGTCATCACCATTG	CAGGAAGGAAGGCTGGAAAAG
IL-1β	GTGGACAACAAAGCTCGTGG	AGCCCGTCAACCTCAAAGAA
IL-6	TGTCTTCTTGGGACTGCTGC	CCAAACCTCCGACTTGTTGA
TNF-α	CACCCACCGTCAAGGATTCA	TTGGCTGGGCAATGAAGAGT
IFN-α	AGACTGGGAGTTGCCTGTGA	GAGGAATCCAGGGCTTTCCAG
IFN-γ	TGCATCTTGGCTTTGTTGCTC	TCCCCTCCATTCACGACATC
MIP-1α	GGTCCAAGAGTACGTCGCTG	GAGTTGTGGAGGTGGCAAGG
RANTES	TCAGCTTGGTTTGGGAGCAA	TGAAGTGCTGGTTTCTTGGGT

IP-10	TACGTCGGCCTATGGCTACT	TTGGGGACTCTTGTCACTGG
